# Organising Concepts of ‘Women’s Empowerment’ for Measurement: A Typology

**DOI:** 10.1007/s11205-018-2012-2

**Published:** 2018-10-11

**Authors:** Lu Gram, Joanna Morrison, Jolene Skordis-Worrall

**Affiliations:** Institute of Global Health, University College London, 30 Guilford Street, London, WC1N 1EH UK

**Keywords:** Women’s empowerment, Critical review, Low- and middle-income countries, International development, Feminism, Political philosophy

## Abstract

Improving the conceptualisation and measurement of women's empowerment has been repeatedly identified as a research priority for global development policy. We apply arguments from feminist and political philosophy to develop a unified typology of empowerment concepts to guide measurement and evaluation. In this typology, empowerment (1) may be a property of individuals or collectives (2) may involve removing internal psychological barriers or external interpersonal barriers (3) may be defined on each agent’s own terms or by external agents in advance (4) may require agents to acquire a degree of independence or require others to ‘empower’ them through social support (5) may either concern the number of present options or the motivations behind past choices. We argue a careful examination of arguments for and against each notion of empowerment reveal fundamental fact-, theory- and value-based incompatibilities between contrasting notions. Thus, empowerment is an essentially contested concept that cannot be captured by simply averaging a large number of contrasting measures. We argue that researchers and practitioners measuring this concept may benefit from making explicit their theory-, fact- and value-based assumptions about women’s empowerment before settling on a single primary measure for their particularly context. Alternative indicators can subsequently be used as sensitivity measures that not only measure sensitivity to assumptions about women’s social reality, but also to investigators’ own values.

## Introduction

Women’s empowerment is widely recognized as a global policy objective (UN General Assembly 2015) and a key component of strategies to promote health and combat poverty world-wide (World Bank 2012; Every Women Every Child 2017). However, development researchers have repeatedly encountered difficulties in constructing indicators for its measurement (Kabeer 1999; Richardson 2018; Malhotra and Schuler 2005; Raj 2017) and have commonly identified improving the conceptualisation and measurement of empowerment as a research priority (Ibrahim and Alkire 2007; Cunningham et al. 2014; Carlson et al. 2015).

In this article, we draw on feminist and political philosophy to clarify debates over the meaning of women’s empowerment and generate a typology for its plural interpretations. We argue for a series of fact-, theory- and value-based judgments inherent in most measures of empowerment that create fundamental incompatibilities between contrasting notions of empowerment. Thus, we argue researchers and practitioners might benefit from selecting a single notion of empowerment as their primary measure in empirical applications and treating other measures as secondary measures testing sensitivity to their own assumptions.

## The Need for a Typology

Existing development discourse has often debated appropriate methods for the measurement of ‘true’ empowerment (Richardson 2018; Cueva Beteta 2006; Klasen and Schüler 2011). A recent review identified three obstacles to improving measures of empowerment (Richardson 2018): (1) Poor integration of theory into the development of indicators (2) Implicit judgment and bias in methods of analysing data (3) Narrow choice of indicators that fails to capture the full scope of the empowerment concept. All of these tasks require researchers to first establish what they mean by ‘women’s empowerment’.

This likely requires judgments of both value and fact—empowerment is a ‘thick ethical concept’ (Williams 1985) that lies in the middle of a continuum between purely value-laden and purely factual concepts (Putnam 2002). For example, when the Fifth Sustainable Development Goal aims to ‘achieve gender equality and empower all women and girls’ (UN General Assembly 2015) or when empowerment is labelled ‘intrinsically valuable’ (Trommlerová et al. 2015), then researchers and policy-makers are directly appealing to its normative aspects. Yet the Fifth Sustainable Development Goal also refers to a factual state of the world that the global community hopes to achieve by 2030. More generally, thick ethical concepts usually involve an inextricable entanglement of facts, values and theories (Putnam and Walsh 2007).

Value-based disagreements are notoriously difficult to settle (Railton 1986). Political philosophers have argued that power and freedom are ‘essentially contested’ (Lukes 2005; Gallie 1956) or ‘irreducibly plural’ (Alkire 2005a) concepts for which widespread agreement exists concerning their practical importance in the real world, while disagreement abounds concerning their proper interpretation. These disagreements stem from entirely reasonable fundamental differences in philosophical standpoints rather than simply differences in linguistic convention, lack of logical clarity, or lack of empirical evidence.^1^

For example, suppose a newly married Nepalese couple wants to have a child. The young wife would like to finish her university education and get a job but also worries about spending sufficient time with her child as a professional woman. The husband proposes to his wife that she forego her education and stay at home to raise the child. We could class his proposal as either empowering or disempowering depending on our fact-, value- and theory-based assumptions. We could class it as disempowering and paternalistic by exposing the woman to risk by leaving her future access to material well-being in her husband’s control. We could class it as caring and empowering, because it unburdens her from having to juggle childcare and further study in a resource-constrained context.

We present this example, not to argue for a single interpretation of empowerment, but to illustrate a situation where we cannot decide on a measure of empowerment without making value-laden choices. Stating that the woman’s situation contains both aspects of empowerment and disempowerment does little to clarify how we should assess her, since most social situations can be classified as either empowering or disempowering depending on one’s values and assumptions. Neither can we create an aggregate index of both concepts, since their respective arguments directly contradict one another. Rather, we need to make a choice.

Feminist and political philosophers have uncovered many such situations, which we will describe. Nevertheless, development researchers have often aggregated a large number of divergent empowerment concepts into a single index without fully considering whether the arguments for each component measure are logically compatible (Alkire et al. 2013; Ibrahim and Alkire 2007). Thus, we believe there is a need for an accessible framework that organises contrasting conceptualisations of empowerment and highlights situations where these contrasts cannot be aggregated.^2^

## A Typology of Concepts

**Fig. 1:**
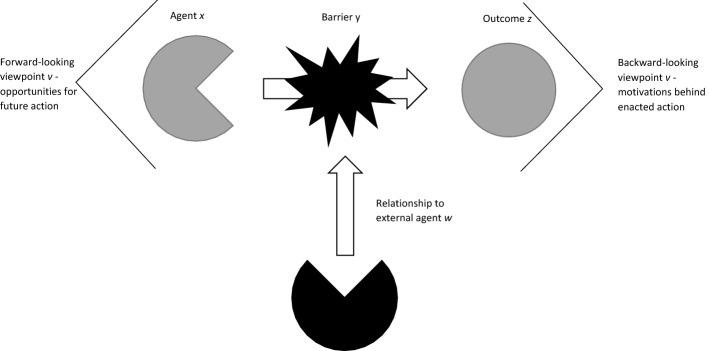
Conceptual framework for empowerment: Agent x is empowered if they are able to overcome barrier y to achieving outcome z, possibly posed by external agent w. The viewpoint v concerns whether we are primarily concerned with future opportunities for achievement (forward-looking) or past motivations for enacted actions (backward-looking)

Figure 1 shows an overall framework for defining empowerment, adapted from a seminal formula by Gerald MacCallum (1967):

Whenever the freedom of some agent or agents is in question, it is always freedom from some constraint of, restriction on, interference with, or barrier to doing, not doing, becoming, or not becoming something … agent x is (is not) free from y to do (not do, become, not become) z (p. 314).

An *empowered* agent *x* possesses the ability to reach an outcome *z* when faced with a barrier *y. Empowerment* is the process through which an agent becomes empowered. When we measure an agent’s *level of empowerment*, we are measuring the extent to which this agent is empowered.

This framework unifies many disparate conceptions of empowerment. By varying the values of *x, y* and *z*, we obtain distinct concepts of empowerment. Additional axes of variation are represented by variables *v*—the viewpoint from which we assess agent abilities-and *w*—the relationship to external agents. *v* refers to the extent to which we consider empowered agents to have many options available or to feel highly satisfied with the choices they have made. *w* refers to the extent to which empowered agents are free of others’ interference or blessed with others’ support.

The contrasting aspects of each variable are summarised in Table 1. In the following sections, we discuss each distinction in detail and delineate the reasons (1) why the distinction matters (2) what it entails for measurement.

**Table 1:** Description of theory, fact, and value-based judgments inherent in most conceptualisations of empowerment

Judgment	Description	Why the distinction matters	What it means for measurement
Individual versus collective empowerment	Is the agent of empowerment an individual or is it a group?	Individual empowerment may be either aligned with, independent of, or opposed to collective empowerment, depending on our conceptualisation of the two notions	When collective empowerment is based on limits to individual freedom, we cannot easily combine individual and collective measures into one score
Internal versus external barriers to empowerment	Are the barriers to empowerment internal to the agent or external to the agent?	Internal notions of freedom may be appropriated for freedom-limiting purposes Internal notions of freedom reflect ability to cope with oppression rather than lack of oppression itself External notions of freedom ignore false consciousness External notions of freedom ignore second-order desires External notions of freedom reflect masculinist bias	When external freedom is endorsed, precisely because it is not internal freedom (e.g. internal freedom measures coping ability rather than empowerment), then we cannot easily interpret an average score of both internal and external measures
Forward-versus backward-looking viewpoint	Should empowerment be assessed based on the motivation leading up to an outcome or on opportunities for future outcomes?	Too much choice can demotivate decision-making Forward-looking freedoms do not entail exercise of freedom Backward-looking freedoms do not entail availability of opportunity	When a loss of opportunity leads to greater life satisfaction, the two types of freedom are mutually opposed; in such a case, it is difficult to interpret an average both types of freedom
Direct versus indirect freedom	Do agents’ need to be directly involved in realising their own outcomes for it to constitute empowerment or can others make decisions on agents’ behalf?	Indirect freedom may encourage dependency on others Direct freedom expects too much from self-reliance Direct freedom reflects masculinist bias Indirect freedom may marginalise women’s voices	Since most acts of social support necessarily simultaneously entail a degree of dependency, the values of direct and indirect freedom are usually opposed; in such a case, it is difficult to interpret an average of both types of freedom
Subjective versus objective interests	Should the goal of empowerment be determined by agents themselves or by independent experts?	Notions of objective interest may be paternalistic and discredit agent’s own ability form a conception of the good Notions of subjective interest may ignore false consciousness	When an agent’s subjective interests are the opposite of their objective interest, then we cannot easily interpret an average of their ability to achieve both types of interest

## Type of Agent *x*: Individual or Collective

### Why the Distinction Matters

The first judgment in MacCallum (1967)‘s formula concerns the choice of agent, *x*. While the term ‘women’s empowerment’ clearly signifies a focus on women (Richardson 2018), it is ambiguous whether it refers to the empowerment of individual women (*x *=‘an individual woman’) or the empowerment of women collectively (*x *= ‘a collective of women’) as an interest group (Jónasdóttir 1988). This matters because collective empowerment has a highly contested relationship to individual empowerment.

#### Collective Empowerment May Increase in Individual Empowerment

Community psychologists have often seen individual empowerment as positively related to collective empowerment (Wallerstein and Bernstein 1994). If we conceptualise collective and individual empowerment as the ability of groups and individuals to achieve collective and individual goals respectively, then collective empowerment arguably increases with individual empowerment, as long group and individual goals are aligned. For example, all-female micro-credit groups have been observed to spontaneously mobilise to prevent violence against women in their community (Sanyal 2009). The ability of the whole group to prevent violence against women arguably increases in group members’ individual ability to support women facing violence.

#### Collective Empowerment May be Independent of Individual Empowerment

A positive relationship between individual and collective empowerment requires a conception of individual empowerment that includes individual possession of interpersonal capabilities, since the ability of groups to achieve collective outcomes usually depends as much on the quality of member relations as on individual abilities. Group dynamics, social identity, and group leadership all play a major role in aggregating individual capabilities into collective power (Radke et al. 2016; Freeman 1972; Cohen and Bailey 1997). A major study of small group effectiveness involving 192 groups found empirical evidence for a collective intelligence factor, a ‘c factor’, which depended more on average social sensitivity of group members than individual intelligence (Woolley et al. 2010). Large groups face high transaction costs when coordinating action across geographical areas which prevent collective interests from translating into collective action (Heckathorn 1996). A 1983 Gallup poll showed 40% of US residents believed there might be nuclear war by 1998 and 70% believed they would not survive it, but only a very small minority engaged in collective protest (Hornsey et al. 2006).

Even so, individual capability is not guaranteed to aggregate straightforwardly into collective capability. Social capital researchers have frequently commented on the need to distinguish between social capital as an individual benefit through social connections, or a collective resource that benefits communities or societies (Poortinga 2006; Portes 2000; Kawachi et al. 2004). Even if many women individually report high degrees of social support, this does not necessarily entail women collectively are empowered, if these women all draw on social support from the same person.

Similarly, Cohen (1983) noted how widespread individual capability may be compatible with a complete lack of collective capability: A policy that reserves 1% of civil servant jobs for local women might empower women individually, since they all have an individual opportunity to be part of the select 1%, but their collective capability to enter government service has not altered noticeably. Tokenistic offers of elite status to a small number of oppressed people may even be used to discourage protest and maintain hierarchical power structures (Wright and Taylor 1999).

In all the above cases, collective empowerment may be largely independent of individual empowerment.

#### Collective Empowerment May Decrease in Individual Empowerment

When individual and collective goals are not aligned, then collective empowerment may even be opposed to individual empowerment. A common theme in social anthropology (Rao 2005), team psychology (Barker 1993), and sociology (Bauman 2013) is the tendency for groups to empower themselves to achieve collective goals by restricting the power of their members. For example, Rao (2005) observed how the Indonesian ideology of *svadaya gotong royong* promoted community capacity by requiring members to contribute to collective projects lest they be labelled ‘unpatriotic’ and subject to social sanctions by fellow members. All-female, joint liability microcredit groups have been lauded for their ability to achieve high levels of loan repayment through the use of shame, sanction and pressure to conform (Banerjee 2013). Yet researchers have commented on their potential to generate excessive peer pressure through verbal harassment, public shaming and confiscation of assets leading to reported incidents of stress and even suicide in over-indebted borrowers (Rahman 1999; Ahmed et al. 2001). The paradoxical ability of collective empowerment to restrict individual freedom has been polemically labelled ‘The Tyranny of Participation’ (Cooke and Kothari 2001).

### What this Means for Measurement

When collective empowerment is independent of or opposed to individual empowerment, we cannot obtain valid measures of collective empowerment from measures of individual empowerment. When collective capacity derives its power from social control of individuals, we cannot easily combine collective and individual empowerment into an aggregate index.

## Type of Barrier *y*: Internal or External

### Why the Distinction Matters

Berlin (1959) introduced a well-known distinction between ‘positive’ and ‘negative’ freedom, where the ‘positive’ notion referred to a form of self-realisation—people acting according to their authentic principles and avoiding the temptations of their irrational impulses—and the ‘negative’ notion referred to a condition of being free from interference, coercion or restraint from other people. MacCallum (1967) categorised Berlin’s distinction in terms of the choice of barrier y. In positive freedom, agent x attempted to overcome internal, mental barriers. In negative freedom, the barriers were external and interpersonal. Over time, political and feminist philosophers have raised numerous arguments for and against either concept.

#### Internal Notions of Empowerment May be Appropriated for Freedom-Limiting Purposes

Berlin (1959) was strongly critical of internal notions of freedom, arguing they were open to abuse by totalitarian states who could restrain citizens’ liberties by claiming they were freeing them from their lower impulses. He argued a prisoner in an isolation cell could be considered free by such definitions, if their actions continued to accord with their guiding values. For example, studies have shown that judgments of the authenticity of conflicting desires are often conveniently made to fit viewers’ own political values. When American undergraduate students were presented with a hypothetical case of a devout Christian who felt repressed homosexual impulses, conservative participants located his ‘true self’ in his Christian principles, while liberal participants located it in his homosexual impulses (Newman et al. 2013).

#### Internal Notions of Empowerment Reflect Ability to Cope with Oppression Rather than Lack of Oppression Itself

Feminist psychologist Riger (1993) castigated internal notions for promoting ‘the *sense of* empowerment’ (p. 281) over actual power, making the political personal, and supporting the status quo. Watts and Hipolito-Delgato (2015) rhetorically asked about the feasibility of ‘thinking ourselves to liberation’. Kitzinger (1991) argued internal notions of power blamed victims of rape and other forms of abuse for their victimhood, reinforced the structural drivers of gender inequality, and assumed an ‘individualist myth’ of the ‘free, autonomous, self-fulfilled and authentic woman’ (p. 124). Berlin (1959) similarly felt internal notions of freedom reflected an individual’s ability to cope with oppression rather than an objective lack of oppression itself. By appropriately reducing individual aspirations, any individual could claim to attain complete internal freedom. He called this the ‘doctrine of sour grapes’ based on an Aesop fable wherein a fox, unable to reach a set of grapes, exclaimed that these were sour anyway.

#### External Notions of Empowerment Ignore Issues of False Consciousness

Taylor (1979) sharply criticised external notions of freedom for ignoring internal, mental obstacles, arguing the very idea of states protecting individual freedom depended on a community that valued such freedoms. Following a long tradition of thinkers from Marx to Lukes, he argued that action guided by ‘fear, inauthentically internalised standards, or false consciousness’ (p. 215) should be considered cases of unfreedom, because individuals often underwent socialisation processes that made them accept oppressive norms and values as natural and inevitable features of reality (Lukes 2005; Freire 1972; Femia 1987; Eyerman 1981). Insufficient critical awareness thus became a key barrier to engage in action to change one’s circumstances (Taylor 1985). For example, Busby’s (1999) ethnography of a South Indian fishing village revealed how local women often saw domestic violence as a simple ‘force of nature’ that emerged when men lost their temper. This rendered men’s active role in perpetrating domestic violence invisible and made it difficult for women to imagine situations where such violence did not happen.

#### External Notions of Empowerment Ignore Second-Order Desires

Dworkin (1988) pointed out that individuals have both first-order desires for substantive achievements and second-order desires, which are desires about desires. For example, a married woman with conflicting feelings about her abusive husband might have a first-order desire to please her husband and a second-order desire to stop being a pleaser. In Dworkin’s (1988) conception of autonomy, individual capacities for emotional and behavioural self-control were central to freedom. Such capacities cannot be captured using external notions of empowerment.

#### External Notions of Empowerment Reflect Masculinist Bias

Feminist philosophers argue certain external conceptions of power are based on notions of interpersonal dominance, or ‘power-over’ other people (Wartenberg 1992). Such notions may embody implicitly hierarchical, masculinist values of domination and control (Wartenberg 1992). Their realisation may also merely alter the identity of people who hold power leaving the underlying structures of hierarchy and inequality intact (Irigaray 1985). Feminist philosophers have instead advocated for conceptions of power as an inner mental or spiritual strength (Starhawk 1987) allowing agents to overcome self-hatred, inferiority complexes, or internalised homophobia and sexism (Rowlands 1997; Calvès 2009; Kitzinger 1991). This alternative, internal notion of empowerment is called ‘power-from-within’ (Starhawk 1987).

### What this Means for Measurement

When any of the aforementioned arguments are believed to have merit, it is hard to argue for using an internal measure of empowerment to proxy for external empowerment or vice versa.^3^ Indeed, many arguments in favour of internal empowerment vigorously oppose external empowerment and vice versa. This makes it difficult to create aggregate indices of internal and external notions. For example, the concern that internal notions of empowerment measures ability to cope with oppression rather than alleviation of oppression itself hardly diminishes if we take a weighted average of internal and external measures. The potential for external notions of empowerment to perpetuate values of dominance and hierarchy does not diminish if researchers simply complement an external measure with an internal measure. If we theorise individual selves as having second-order desires, then external notions of freedom may be tangentially related to empowerment.

## Type of Viewpoint *v*: Forward- or Backward-Looking Freedom

### Why the Distinction Matters

Taylor (1979) distinguished between ‘exercise freedom’, where the deciding factor was whether individual actions were motivated by their own or other people’s values and goals, and ‘opportunity freedom’, which was determined by the number of options available to a person. Other names for this distinction include ‘episodic power’ versus ‘dispositional power’ (Cobb 1984); ‘enacted’ versus ‘potential’ power (Provan 1980); ‘process’ versus ‘opportunity freedoms’ (Sen 1993). We call this variable ‘viewpoint’ and distinguish between ‘forward-looking freedoms’ that look ahead to see what opportunities lie in the future and ‘backward-looking freedoms’ that assess agents’ motivation behind past actions. Another formulation of backward-looking freedoms was offered by Drydyk (2013) who asserted that ‘agency’ really indicated the extent to which a person’s activities were ‘owned’ by themselves and ‘alienated’ from themselves.

#### Too Much Choice can Demotivate Decision-Making

It may seem counterintuitive that forward- and backward-looking notions can conflict with one another, as increased choice ought to facilitate satisfaction of individual preferences. However, this is not necessarily the case in practice; situations with ‘too much choice’ may demotivate individuals, paralyse decision-making and make people more prone to regret their choice, whatever they finally choose (Iyengar and Lepper 2000). Increased availability of opportunity may also increase individual aspiration (Beaman et al. 2012; Genicot and Ray 2017), potentially leaving individuals no more satisfied with their choices than before their opportunities expanded.

#### Forward-Looking Freedoms Do Not Entail Exercise of Freedom

Forward-looking notions of freedom are criticised for considering individuals to be free regardless of whether they actually exercise their freedoms (Taylor 1979). Political theorists have argued that freedom is a habit that individuals need to inculcate through repeated participation in decision-making (Gerber et al. 2003). Other philosophers have asserted that modern states subtly limit individual freedom by providing legal opportunities for political power while simultaneously socialising citizens to stay disengaged (Rose 1999). Feminist theorists have often highlighted the fact that Muslim women in South Asia are legally entitled to a share in parental land under Islamic law, but usually refrain from exercising these rights for fear of losing social support from their family members (Kabeer 1999; Agarwal 1994).

#### Backward-Looking Freedoms Do Not Entail Availability of Opportunity

Backward-looking freedoms are criticised for considering individuals free, even if they only have one option to choose from, as long as this option happens to align with their values and goals (Bavetta 2004). This is potentially dangerous, since a major value of freedom rests in its ability to guarantee flexibility in the face of unexpected outcomes (Carter 1995). Even if a person is fully satisfied with their present life, they may not be able to accurately forecast future life satisfaction. Without the opportunity to conduct ‘experiments in living’ (Carter 1995) whereby they get to experience the outcomes of their own choices, they may find themselves unable to escape a life trajectory they no longer value.

### What this Means for Measurement

In situations where women’s forward-looking freedoms are potentially opposed to their backward-looking freedoms, we cannot assume that one measure of empowerment acts as a good proxy for the other measure. In such situations, it also becomes difficult to defend an aggregate index of both measures. For example, if a medically trained woman gives up a career as a doctor because her husband forbids her to work, she may report that her opportunities for employment reduced to zero, but she is satisfied with being unemployed, as she genuinely wants to please her husband. If we believe that the opportunity to conduct ‘experiments in living’ is critical to empowerment, we cannot simply consider her ‘half empowered’, since the opportunity to even try living as a doctor has been denied her.

## Relationship to External Agent *w*: Direct or Indirect Freedom

### Why the Distinction Matters

This axis of variation only applies to concepts of empowerment where the barrier involves an external agent. A recurring debate in the theory of empowerment is whether empowerment should be defined in terms of ‘direct’ or ‘indirect’ freedom (Iversen 2003; Sen 2005; Alkire 2008). Direct freedom refers to direct involvement in realising one’s own goals, while indirect freedom allows for third-party intermediaries to help realise one’s goals and values, possibly without one’s full awareness. This distinction has also been referred to as the difference between ‘effective power’ and ‘choice-mediated control’ (Iversen 2003), ‘effective power’ and ‘procedural control’ (Sen 1985), ‘personal control’ and ‘proxy control’ (Bandura 1997), or ‘policy control’ and ‘implementation control’ (Kabeer 1997).

#### Indirect Freedom May Encourage Dependency on External Agents

Opponents of indirect freedom tend to advocate for greater independence in external relationships (Kitzinger 1991) to protect agents from perceived risks of exploitation and conflict (Kabeer 2001). For example, Iversen (2003) argued that indirect freedom was disempowering and paternalistic, because it deprived agents of opportunities to cultivate self-reliance and decision-making skills. Such agents became dependent on others to realise their own goals and needed to continually ingratiate themselves with their supporters to extract desired favours. Iversen argued such notions of empowerment could be used to legitimise the status quo where a male patriarchal household head ‘supported’ female household members by ruling over them.

#### Direct Freedom Expects Too Much from Self-Reliance

Supporters of indirect freedom tend to recast dependency as social support and view direct freedom as disempowering, because it burdens agents with the responsibility of realising goals and values without assistance from others. Sen (1985) argued that indirect freedom necessarily formed part of people’s experience of freedom, since the interdependent nature of society made it impossible for any single person to be fully self-reliant. For example, Kabeer (1997) noted how urban Bangladeshi women did not experience leaving violently abusive husbands as empowering, because they felt socially isolated and vulnerable to harassment and assault by men in public.

#### Direct Freedom Reflects Masculinist Bias

Feminist theorists criticised concepts of empowerment based on individualism and ‘a preference for traditionally masculine concepts of mastery, power, and control over traditionally feminine concerns of communion and cooperation’ (p. 279) (Riger 1993) and called for alternatives to an obsession with autonomy and independence to the point of ‘autonomy fetishism’ (Khader 2015). Riger (1993) argued that traditional notions of power had marginalised behaviour belonging to the communal, expressive, ‘feeling’ realm and had led feminists to punish women for making relationships and connections central to their lives.

#### Indirect Freedom May Marginalise Women’s Voices

Kitzinger (1991) argued that critiques of ‘power-over’ as a masculinist concept reinforced women’s oppression by perpetuating the myth that the two sexes held separate spheres of competence, which had been invoked by philosophers ‘from Aristotle through Rousseau to Freud’ to argue for ‘women’s different and (implicitly and explicitly) inferior understandings of political issues like justice, ethics and power’ (p. 115). Other feminists cautioned that intransigent conflicts of interests across societal divides are not easily be solved through a naïve emphasis on harmony (Guijt and Shah 1998) and that invocations of ‘community’, ‘family harmony’ and the pressures of male ‘breadwinners’ carry their own risk of silencing marginalized voices and hiding power differentials within households (Guijt and Shah 1998; Batliwala 2007; Folbre and Nelson 2000).

### What this Means for Measurement

Arguments for direct freedom often vigorously oppose indirect freedom and vice versa. If we genuinely believe that indirect freedom promotes dependency in unequal power relations and marginalises women’s voices, then it does not make sense to consider an economically dependent married woman ‘half-empowered’, because she receives money from her earning husband. Similarly, if we sincerely believe that direct freedom reflects a masculinist bias with independence over communion and imposes burdens on women to escape oppression through self-help, then it does not make sense to consider an economically independent woman ‘half-empowered’, because she earns her own income. In such cases, measuring one as a proxy for the other or computing an average of the two measures does not seem advisable.

## Type of Outcome *z*: Subjective or Objective Interests

### Why the Distinction Matters

The final variable in MacCallum’s formula *z* denotes the outcome that an agent can achieve. The specification of *z* is important, since ‘some life choices have larger implications for women’s agency than others’ (Richardson 2018). For example, repairing potholes in the road can hardly be said to disempower local women, even if it restricts their ‘freedom’ to fall into one, because such achievements are not relevant (Taylor 1985).

An enduring debate over the choice of *z* concerns whether the promotion of individuals’ ‘subjective interests’ or their ‘objective interests’ should be considered a form of empowerment (Jónasdóttir 1988). Theorists have been divided over whether every individual should determine the relevant outcome *z* according to their own unique standards (‘subjective interests’) or whether it is possible to discern at least some universally acceptable domains of life through careful reasoning by a group of experts (‘objective interests’) (Jónasdóttir 1988).

For example, calls by Western human rights activists to take action against female genital mutilation in Sub-Saharan Africa have sometimes backfired and made local women more confident about asserting their own ‘cultural obligations’ (Althaus 1997) to continue the practice. In such situations, women’s ability to further their objective interest of combating female genital mutilation has decreased, while their ability to further their subjective interest of preserving tradition has arguably increased.

Proponents of subjective interests have argued that expert-driven agenda-setting ignores women’s perceptions of their own empowerment (1997) and comes with risks of paternalism (Khader 2011). Proponents of objective interests have argued that respondent-driven agenda-setting ignores individual information constraints (Paasche-Orlow and Wolf 2007) and psychological biases (Thaler and Shefrin 1981). These arguments mirror the debate over internal versus external barriers to empowerment, thus we will not repeat them here.

Reliance on subjective interests can sometimes be used by researchers to resolve choices about the other variables *x*, *y*, *v* and *w* in our framework. For example, researchers could ask women themselves to choose whether they prefer direct or indirect freedom and score them accordingly. Indeed, Chirkov et al. (2003) argued autonomous individuals choose to be dependent on people who are perceived as supportive and independent of people perceived as coercive. Pettit (1996, 1997) argued external interference only limits freedoms if it is perceived as illegitimate. Although this position may seem to help researchers avoid hard choices, it does require them to make a formidable commitment to women’s subjective interests.

Consider the case of female genital mutilation. Suppose woman A believes women should be forced to undergo the procedure to achieve the collective goal of preserving traditional culture, while woman B believes women should be individually free to make their own choice. An extreme subjectivist viewpoint would commit us to view both women as equally empowered if A was forced to undergo the procedure, while B chose not to do it, because both women had their wishes realised, even if those wishes were based on opposite values.

### What this Means for Measurement

Unless researchers have good knowledge of the preferences of their target population and have tailored their measurement of empowerment accordingly, it is likely that women’s objective interests, as determined by outside agents, will differ from their subjective interests. In such cases, it is unlikely that researchers can obtain accurate information about women’s ability to achieve their subjective interests from measures of empowerment in the objective realm and vice versa for objective interests. When subjective and objective measures of empowerment are opposed to one another, we can usually not take a simple average of the two.

## Classification of Existing Indicators

Table 2 displays commonly used measures of women’s empowerment in international development along with a classification of their location in our typology. We have assessed each indicator based on what it directly measures rather than what it may proxy for or what it may measure in practice, once cultural, social and cognitive biases enter the picture.^4^

**Table 2:** Classification of example measures of empowerment

Measure	Classification method	Agent	Barrier	Interest	Direct/indirect freedom	Forward/backward-looking
Who has the final say over household purchases?	Woman has the final say alone or jointly with others = empowered, otherwise disempowered	Woman herself	External	Objective interests	Indirect freedom	Forward-looking
Who has a say over household purchases? Would you be able to have a say over household purchases if you wanted to?	Woman has a say alone or jointly with others = empowered, otherwise disempowered	Woman herself	External	Subjective interests	Indirect freedom	Forward-looking
Who decided on past household purchases?	Woman decided alone or jointly with others = empowered, otherwise disempowered	Woman herself	External	Objective interests	Indirect freedom	Backward-looking
Who usually executes decisions on household purchases?	Woman executes decisions alone = empowered, otherwise disempowered	Woman herself	External	Objective interests	Direct freedom	Forward-looking
The Relative Autonomy Index	Higher levels of internal motivation = more empowered, higher levels of external motivation = less empowered	Woman herself	Internal and external	Subjective interests	Either—respondent chooses	Backward-looking
Specific self-efficacy to do *X*	Higher levels of confidence = more empowered, otherwise less empowered	Woman herself	Internal	Objective interests	N/A	Forward-looking
Generalised self-efficacy	Higher levels of confidence = more empowered, otherwise less empowered	Woman herself	Internal	Subjective interests	N/A	Forward-looking

### Decision-Making Measures

The Demographic and Health Surveys (Kishor and Subaiya 2008) routinely ask married women of reproductive age ‘who in your family usually has the final say on’ a variety of domains from household purchases to visits to family and friends in countries around the world. The response options typically include the woman herself, the woman jointly with her husband, the woman jointly with other household members, her husband only, or other household members only. As observed by Kabeer (2001), some researchers consider women empowered only if they make decisions solely on their own (De Brauw et al. 2014), while others consider them empowered if they participate in decisions whether on their own or jointly with others (Singh et al. 2014).

For this measure, the agent in question is clearly a single individual rather than a collective. The barriers to her empowerment are external household relations.^5^ Usually these questions are used to indicate empowerment for women without evidence that women themselves feel the domains being queried are important (Ahmed et al. 2010; Shroff et al. 2009), which bespeaks a targeting of objective interests pre-determined by outside experts. The measure takes a forward-looking viewpoint, because it focuses on what decisions the respondent is able to influence, i.e. ‘have the final say on’, rather than what decisions the respondent tends to exercise in practice. For example, a mother-in-law who is utterly uninterested in supervising her daughter-in-law’s cooking activities will still be counted as empowered in the domain of food preparation if she has the authority to intervene, should she wish to do so. Finally, the measure places a strong emphasis on indirect freedom, since women do not need to directly involve themselves with executing decisions in order to count as empowered.

The target concept of this measure is quite sensitive to adjustments in the wording and scoring of survey questions. The measure could be altered to focus on overcoming internal, mental barriers, if it was reworded to ‘How confident do you feel that you can control your own spending?’ The emphasis on women’s objective interests could be moved towards subjective interests by using qualitative research to determine if the domains being asked were considered relevant to the respondents themselves (Schatz and Williams 2012), or by asking women in the survey itself whether they wanted to make decisions in the domain being queried (Ibrahim and Alkire 2007). The forward-looking viewpoint could be changed to a backward-looking viewpoint by asking ‘who decided on past household purchases’. The focus on indirect freedom could be changed to an emphasis on direct freedom by asking ‘who usually executes household purchases’ and only scoring the respondent as empowered if she alone does this.

### Self-Efficacy

Bandura’s (2001) concept of self-efficacy is an oft-proposed (Alkire 2005b; Schuler et al. 2010; Alkire 2008; Malhotra and Schuler 2005) and used (Upadhyay et al. 2014; Swendeman et al. 2009) measure of women’s empowerment in international development. A typical question asks women about their perceived skill controlling a specific behaviour, such as condom use (Albarracín et al. 2004). Self-efficacy is itself one of many possible measures of ‘psychological empowerment’ (Zimmerman 1995; Johnson et al. 2005) that focus on individual ability to regulate their emotions and control their own behaviour.

Bandura saw his measure of self-efficacy as measuring the ability of a single, integral self in overcoming mental barriers internal to itself (Bandura 1999). The barriers to empowerment are clearly internal, as individuals are considered empowered, when they feel empowered, regardless of the presence of external constraints on their behaviour. Individuals are not classed as empowered if their environment changes, unless they themselves perceive their environment to have changed. For example, a socially anxious woman who lacks confidence to attend university as a mature student, because she fears ridicule from other students is disempowered, even if no-one has any intention of actually ridiculing her. Measures of self-efficacy are typically forward-looking, since they ask respondents what they can do rather than what motivated them to do what they did in the past. The distinction between direct and indirect freedom is not applicable, as self-efficacy is an internal notion of empowerment.

Bandura emphasised that ‘generalised self-efficacy’ does not exist, because self-efficacy is a domain-specific construct, which only attains adequate predictive validity when one examines ‘microrelations at the level of particular activities’ (Bandura 1997); for example, a sports player’s perceived self-efficacy in winning a particular game against a particular opponent in a particular setting. Other researchers have argued for the validity of questions on generalised self-efficacy based on factor analyses of psychometric data (Judge et al. 2002; Scholz et al. 2002). Such questions would ask women to agree or disagree with questions such as ‘I can always manage to solve difficult problems if I try hard enough’ or ‘I can handle whatever comes my way’ (Scholz et al. 2002).

If researchers measure women’s empowerment using a domain-specific measure of self-efficacy, e.g. exercise self-efficacy (Strecher et al. 1986), then they implicitly operate with a notion of objective interest, since they have decided in advance on an outcome for individuals to achieve. If researchers use a measure of generalised self-efficacy, then they work with a notion of subjective interest, since respondents are likely to imagine outcomes that they feel are important to themselves.

### The Relative Autonomy Index

Ibrahim and Alkire (2007) proposed the use of the Relative Autonomy Index from Self-Determination Theory (Deci and Ryan 2000) as part of a suite of internationally comparable indicators on women’s empowerment. This index has been used in a range of evaluations from the impact of participatory women’s groups on women’s empowerment (Gram et al. 2018a, b) to the impact of health clubs on the empowerment of tribal youth (Sarkar et al. 2017). It also forms part of the Women’s Empowerment in Agriculture Index (Alkire et al. 2013), which has been used in Bangladesh (Sraboni et al. 2014), Ghana (Malapit and Quisumbing 2015) and Nepal (Malapit et al. 2013, 2015).

The index contains a series of structured sections each starting with a framing question followed by questions about individual motivations to carry out or refrain from carrying out activities in a particular domain. For example, a woman might be asked if she performs work inside the house, outside the house or both followed by questions on whether she performs the type of work she has mentioned because she wants to, because it is personally important, because she will get into trouble with others otherwise, or because she is afraid of others’ ill-judgment. She is asked to agree or disagree with each statement on a four-point Likert scale and scored according to the extent to which her motivations reflect greater internal relative to external motivation (Gram et al. 2017; Vaz et al. 2016).

The tool clearly employs a backward-looking viewpoint, as it asks respondents about the motivations behind their actions rather than the range of potential future actions open to them. It includes both internal and external barriers to action. Items such as ‘I do this work because I will get punished otherwise’ measure external barriers, because the presence or absence of an external threat decides women’s level of empowerment (Deci and Ryan 2000). Items such as ‘I do this work because I want people to like me’ are meant to reflect an internal barrier, where fears of self-punishment through feelings of rejection and low self-esteem pressure the respondent to ignore their own authentic values and goals (Deci and Ryan 2000). In contexts where respondents’ well-being and survival depend crucially on being liked by others, the implied motivation of agreeing with this item may be fear of external punishment rather than any form of self-punishment (Gram et al. 2017). In such a case, the index targets purely external barriers to empowerment.

The tool favours respondents’ own subjective interests over any notion of objective interest, since women are directly scored on whether they themselves consider their own activities valuable. If women are unable to participate in household decisions in a domain they will still be counted as empowered, if they appreciate being able to leave such decisions to others. If women feel forced to participate in household decisions by other family members, they are counted as disempowered. Finally, the tool takes a subjectivist position that favours neither direct nor indirect freedom. If women choose to depend on others, then they score just as highly as if they chose to be independent. Women are disempowered if they are forced to be either independent or dependent on others against their will.

### Women’s Collective Empowerment?

Many measures of women’s empowerment include a dimension on group participation or connectedness to social networks (Ibrahim and Alkire 2007; Alkire et al. 2013), but such measures view the agent of empowerment in individual rather than collective terms. Similarly, measures of women’s collective efficacy measure their own individual perceived ability to take collective action (Kuhlmann et al. 2014). Thus, these do not constitute truly collective measures of empowerment; as discussed in our section on collective empowerment, we often cannot aggregate individual measures of empowerment to obtain an accurate picture of collective empowerment.

Measures of community mobilisation (Lippman et al. 2016), community-level social capital (Grootaert 2004; Pronyk et al. 2008; Agampodi et al. 2015), community capability or capacity (Paina et al. 2016; Underwood et al. 2013), collective efficacy (Sampson et al. 2002), sense of community or social cohesion (Kramer et al. 2011; King et al. 2010) could potentially be adapted to measure women’s collective empowerment by treating the set of women living in a particular locality as a ‘community of interest’ (Laverack 2004). However, no researchers have done so in a low- and middle-income context to our knowledge.

## Discussion

### Defining Women’s Empowerment

In this article, we described a typology for classifying concepts of women’s empowerment in terms of fact-, theory- and value-based distinctions implicit in most choices of indicators. We present our typology as a pragmatic tool for researchers and policy-makers^6^ to provide greater clarity on the meaning of empowerment; short, broad definitions based on terms such as ‘choice’, ‘opportunity’, ‘agency’ or ‘autonomy’ often turn out just as unclear and ambiguous as the original ‘empowerment’ concept.^7^

For example, a definition of empowerment as ‘the expansion of agency’ (Ibrahim and Alkire 2007) shifts the question of what ‘empowerment’ means to a question about what ‘agency’ means. A definition of agency as the ‘Ability to identify goals and act upon them’ (Richardson 2018) raises questions about what distinguishes ‘empowerment’ from a general ability to do anything. A definition of empowerment as ‘the process by which those who have been denied the ability to make strategic life choices acquire such an ability’ (Kabeer 1999) leaves open how complex processes of ‘bargaining and negotiation, deception and manipulation, subversion and resistance’ (p. 438) (Kabeer 1999) should be classified in terms of women’s ability to ‘make strategic life choices’.

As a result, the value-laden nature of choices involved in deciding on one conceptualisation of empowerment over another has been partially obscured and researchers have tried to argue for measures of empowerment that target multiple conceptualisations at once, even if these potentially conflict with each other in their assumptions.^8^ For example, Amartya Sen has both argued for notions of agency based on direct and indirect freedom. Sen (1993) stated that ‘the levers of control in one’s own hands (no matter whether this enhances the actual opportunities of achieving our objectives)’ (p. 522) was an important aspect of individual freedom, while Sen (1985) argued that ‘it is often not possible to organise society in such a way that people can directly exercise the levers that control all the important aspects of their personal lives’ (p. 210). Sen (1985) cited an example where a person chooses a medical treatment on behalf of an unconscious friend that aligns with the friend’s values and beliefs as an example of agency enhancement—clearly the opposite of having the ‘lever of control in one’s own hands’.

Thus, we argue that empowerment often cannot be captured by simply averaging a large number of contrasting measures, as these measures may implicitly carry contradictory fact-, theory- and value-based assumptions within them.

### Applying the Typology in Practice

A natural question is to what extent it is necessary to consider all the distinctions in Table 1 in every empirical study. Controversies over generalized versus specific constructs have been characterized as debates between ‘lumpers’—who seek to aggregate narrow concepts into broad concepts—and ‘splitters’—who seek to make fine distinctions by splitting broad constructs into their constituent elements (Judge et al. 2002).

In any one study, a longer and more precise definition of women’s empowerment might be less ambiguous, yet complete clarity can never be obtained for an unobservable construct. More involved definitions can become a Procrustean bed forcing researchers to make needlessly convoluted statements that confuse rather than clarify. Highly specific questions about big concepts may produce more precise, but less valid measurement, as the questions become over-specific for the concept in question (King et al. 2004).

Some researchers have found that seemingly small adjustments to their conceptualisation of empowerment have resulted in large changes in estimated outcomes (Peterman et al. 2015; Richardson 2018). Yet other researchers using economic bargaining theory (Lancaster et al. 2006; Anderson and Eswaran 2009; Basu 2006) or ethnographic research (Mandelbaum 1993; Bennett 1983; Minturn and Kapoor 1993) have conceptualised intra-household power as a single parameter that affects many aspects of life at the same time.

Many concepts of empowerment that are incompatible in theory might turn out to be empirically correlated. In cultural contexts where young, married women are unable to even meet and socialise with other women (Mandelbaum 1993), it is difficult to imagine individual freedoms for women to go out in public to be opposed to their collective empowerment. A woman leaving a physically and emotionally abusive partner has potentially escaped both internal, mental restraints on her life and external, interpersonal restraints at the same time. Objective expert-driven and subjective local agendas for social change may coincide in a development project. Forward- and backward-looking notions of freedom coincide with one another in situations where lack of choice reduces intrinsic motivation.

We suggest a pragmatic approach for choosing indicators depending on study purposes. Policy-makers setting ambitious, global targets for women’s empowerment such as the Sustainable Development Goals (UN General Assembly 2015) cannot feasibly collect pilot data from every relevant context to assess the degree to which different indicators correlate or conflict with one another. Researchers and programme evaluators conducting impact evaluations cannot post hoc decide which of their measures of empowerment they should use. In such situations, we recommend systematically consulting the arguments from Table 1 to decide which are deemed to hold the greatest merit and carry the greatest weight. From this reduced list of arguments, they can then choose the indicator that makes the most sense for their own context as a *primary measure*. If this measure is difficult or expensive to apply directly to their study setting, then they may choose a *proxy measure* if they have strong a priori reason to believe that this measure is highly correlated with their ideal measure. To test the robustness of their results to alternative conceptions of empowerment, they can include other indicators as *secondary measures*. These test sensitivity of indicator choice, not only to assumptions about women’s social reality, but also to investigators’ own values.

Researchers analysing existing datasets such as the Demographic and Health Surveys or the Women’s Empowerment in Agriculture Index (Alkire et al. 2013) should first determine if the available indicators reflect their own fact-, theory- and value-based assumptions about empowerment. This can be ascertained from the classification of common indicators in our previous section (Table 2). If such is the case, they can simply apply the indicators to their research. If such indicators are not available, researchers should reflect on whether any of the arguments for their preferred notion of empowerment can be given up, perhaps because it is not relevant for the local context or because opposing arguments hold greater merit or carry greater weight. If existing indicators can be defended based on the revised set of assumptions, they can again go ahead with their dataset. Otherwise researchers should try to ascertain whether there are grounds for their ideal indicator being empirically related to an existing indicator. If this is the case, they can use the existing indicator as a proxy. If not, the researchers will need to collect primary data. Once, they have chosen a primary indicator, researchers can choose secondary indicators to assess sensitivity to variations in their own fact-, theory- and value-based assumptions.

As an example of our suggested approach, we can consider recent evaluations of the impact of participatory women’s groups on women’s empowerment in rural Nepal (Gram et al. 2018a, b). These have used the Relative Autonomy Index as a primary measure of empowerment, while decision-making questions and a ‘Power Ladder’ measure (Lokshin and Ravallion 2005) were secondary, sensitivity measures (Gram et al. 2018a, b). Gram et al. (2018b) were particularly careful to make explicit the different value-based assumptions underlying different measures of empowerment: the decision-making measure focused on respondent’s objective interests, where the Relative Autonomy Index focused on their subjective interests; the Power Ladder question took a forward-looking view of freedom, where the Relative Autonomy Index took a backward-looking view. In both studies, little evidence for impact was observed regardless of the measure of empowerment that was used, thereby demonstrating robustness of conclusions to variations in fact-, theory-, and value-based assumptions.

### Limitations

A few notions of empowerment have been excluded from our typology. First, some concepts of freedom include barriers that are neither internal nor interpersonal, but impersonal or due to wider societal forces. For example, the phrase ‘freedom from hunger’ signals a concept where the barriers to freedom could stem from an extended drought, a failed harvest, a lack of social protection policies, or a global recession. Studies using very broad definitions of empowerment often implicitly include such barriers in their conceptualisation. Wallerstein’s (1992) definition of ‘powerlessness’ as a ‘lack of control over destiny’ or a ‘generalized lack of control’ allowed her to include poverty and unemployment as indicators of disempowerment in and of themselves. Certain feminist conceptions of the ‘power-to’ concept (Allen 1998) which sought to avoid the negative connotations ‘power-over’ ended up defining ‘power-to’ as a general ability to overcome any barrier and achieve, experience or resist any change in any dimension. From a measurement perspective, such broad definitions allow almost any indicator of positive change to be a direct measure of empowerment; thus, we have not included this sense in our typology.

Second, we limited the scope of our typology to notions of women’s empowerment focused on choice, opportunity or capability. Many political philosophers have discussed the definition of freedom as the acquisition of legitimacy, status or recognition in the eyes of others (Berlin 1959; Morriss 2009; Pettit 1996, 1997). Pettit’s (1996, 1997) republican notion of freedom sees individual freedom as a lack of institutional domination and humiliation. An individual who lived in situations of effective ability to follow their own desires and wishes could still be radically unfree if they were bound by illegitimate institutions. For example, a Black slave living in 18th century America who happened to have a well-meaning master who abstained from using physical punishment and attended to his needs would still be unfree given his master’s institutionally protected right to whip and kill him if he so pleased. We excluded legitimacy-based notions of empowerment from our typology, since their systematic treatment would necessitate analyses of complex social structures of recognition and status that lie outside the scope of this paper. We intend our typology to be used for studying the role of women’s empowerment in international development, not for evaluating the legitimacy of a whole social order. We have similarly avoided discussing relationships between communities and institutions.

Finally, we should emphasise that our typology drew primarily from political and feminist philosophy. A large body of philosophical discussion on the conceptualisation and measurement of empowerment has taken place in other fields such as community development (Billings 2000), community psychology (Saegert and Winkel 1996), critical psychology (Cattaneo and Chapman 2010), and liberation psychology (Prilleltensky 2008). A review of major conceptual issues raised by researchers within such disciplines was outside the scope of this paper, although it might be an avenue of future research.

## Conclusion

In this article, we reviewed major debates over the nature of freedom and power from feminist and political philosophy and applied their arguments to critiquing existing measures of empowerment. By examining the arguments for and against contrasting notions of empowerment, we demonstrated the difficulties of attempting to capture the construct through averaging a large number of contrasting measures. We put forward our typology as a pragmatic basis for future development researchers and practitioners to select primary and secondary measures of empowerment that conform with the particular fact-, theory- and value-based assumptions, investigators feel are justified in their own contexts.

## Footnotes

1 Some researchers argue such disagreements are even an intrinsic *function* of essentially contested concepts, as they create productive and creative conversations about human values (Woolcock 2010).

2 Note that this article will primarily draw on political and feminist philosophy, as a conceptual review of empowerment in other fields such as community psychology—which also have a long history of engagement with the empowerment concept—is outside the scope of this paper.

3 Analogously to the individual case, we can classify notions of collective empowerment into internal or external concepts. Internal notions of collective empowerment would focus on enhancing the capacity of collectives through improved internal organisation (e.g. making members of the collective more co-operative towards one another). External notions would focus on ensuring freedom from interference by outside parties, as in the concept of ‘sovereignty’ (Sarooshi 2003), or establishing equitable partnerships with external agents, such as the State. All the previous arguments for and against internal or external barriers to individual empowerment can be applied to collective empowerment with minor modifications.

4 Space does not permit us to discuss more fully the many statistical and survey administration-related issues in measuring empowerment in practice. Such issues have been extensively covered in related literature (Richardson 2018; Lippman et al. 2016; Raudenbush and Sampson 1999; Alkire et al. 2013; Ewerling et al. 2017; Agarwala and Lynch 2006; Gram et al. 2017; Vaz et al. 2016).

5 Here, we assume the target concept of these questions is an objective level of decision-making power rather than merely respondents’ perceived level of power. This is usually their intended meaning, as researchers have extensively engaged in comparing of responses to such questions between different household members to assess the ‘true’ level of power in a relationship (Jejeebhoy 2002; Allendorf 2007; Godoy et al. 2006).

6 Our typology can be used by any researchers aiming to measure women’s empowerment whether they are creating new indicators or using existing datasets; whether they are using direct survey questions (Alkire et al. 2013), revealed-preference models (Doss 2013), or lab-in-the-field experiments (Almås et al. 2015).

7 This point has also been made in the social capital literature by Almedom (2005) who noted that ‘Brief definitions capture little of the meaning with which constructs such as social capital are imbued’ (p. 944).

8 The same point is made regarding social capital by Kritsotakis and Gamarnikow (2004) who note that the term ‘social capital’ is problematic, because it ‘is pregnant with constituent elements of meaning which sometimes contradict one another’ (p. 44).
